# Relationship Between Muscle-Strengthening Activity and Cause-Specific Mortality in a Large US Cohort

**DOI:** 10.5888/pcd17.190408

**Published:** 2020-08-06

**Authors:** Alpa V. Patel, James M. Hodge, Erika Rees-Punia, Lauren R. Teras, Peter T. Campbell, Susan M. Gapstur

**Affiliations:** 1 Behavioral and Epidemiology Research Group, American Cancer Society, Atlanta, Georgia

## Abstract

**Introduction:**

Muscle-strengthening activity (MSA) has beneficial effects on hypertension, glucose homeostasis, and other health conditions; however, its association with mortality is not as well understood.

**Methods:**

We analyzed data from the Cancer Prevention Study-II Nutrition Cohort (data collection 1982–2014), a prospective US cohort that consisted of 72,462 men and women who were free of major chronic diseases; 18,034 of the cohort died during 13 years of follow-up (2001–2014). We used Cox proportional hazards modeling, controlling for various potential confounding factors, to calculate hazard ratios (HRs) and 95% confidence intervals (CIs) for MSA (none, >0 to <1 h/wk, 1 to <2 h/wk, and ≥2 h/wk) in relation to mortality risk, independent of and in combination with aerobic physical activity.

**Results:**

The association between MSA and mortality appeared to be nonlinear (quadratic trend *P* value, <.001). After multivariable adjustment and comparison with no MSA, engaging in less than 2 hours per week of MSA was associated with lowered all-cause mortality (>0 to <1 h/wk: HR = 0.88, 95% CI, 0.82–0.94; 1 to <2 h/wk: HR = 0.90, 95% CI, 0.84–0.97), but engaging in 2 or more hours per week was not associated with reduced risk (HR = 1.01; 95% CI, 0.92–1.09). Associations were similar but not significant for cancer mortality. Engaging in >0 to <1 hr/wk of MSA was associated with a 19% lower risk (HR = 0.81; 95% CI, 0.71–0.92) of cardiovascular disease mortality, but more time spent in MSA was not associated with reduced risk (quadratic trend *P* value =.005). Associations did not vary by amount of moderate-to-vigorous aerobic physical activity.

**Conclusion:**

Engaging in ≥2 hours per week of MSA was associated with lower all-cause mortality, independent of aerobic activity. Reasons for the lack of association with higher amounts of MSA are unclear. Our findings support recommending muscle-strengthening activities for overall health.

**Summary**

**What is already known on this topic?**

Physical activity guidelines recommend muscle-strengthening activity (MSA) at least twice a week in addition to 150 to 300 minutes per week of moderate-to-vigorous aerobic physical activity. These activities are associated with lowered risk of various cardiovascular disease risk factors and other health outcomes.

**What is added by this report?**

Our study documented an association between moderate amounts of muscle-strengthening activities and mortality risk in a large prospective cohort of older US adults.

**What are the implications for public health practice?**

These results provide additional evidence of the health benefits of MSA and reinforce the value of public health messaging promoting MSA as part of physical activity guidelines.

## Introduction

Public health guidelines state that adults should engage in 150 to 300 minutes of moderate-intensity aerobic physical activity or 75 to 150 minutes of vigorous-intensity activity per week and muscle-strengthening activity (MSA) at least twice a week (1). Substantial evidence indicates that moderate-to-vigorous physical activity (MVPA) is associated with lowered risk of all-cause mortality and various chronic diseases, including cardiovascular disease, type 2 diabetes, and various types of cancer (1). In contrast, although MSA is associated with improved glucose homeostasis and lower risk of various cardiovascular disease risk factors (eg, hypertension), sarcopenia, and musculoskeletal disorders (1–3), its association with mortality has been less studied (4,5).

A recent pooled analysis of 11 cohort studies that included a total of 80,306 adults aged 30 or older with 5,763 deaths found that participation in any MSA was associated with a 23% reduction in risk of all-cause mortality (HR = 0.77; 95% CI, 0.69–0.87) (4). In that pooled study, MSA was also associated with cancer mortality, but not cardiovascular disease mortality. Two key limitations noted by the authors of that analysis were that their MSA measure had a 4-week time frame with no information on longer-term engagement and insufficient power for some analyses. Another meta-analysis and systematic review of 11 published studies examined approximately 370,000 adults aged 18 to 75 with a mean follow-up of less than 9 years (5). That analysis showed that engaging in any MSA was associated with a 21% reduction in mortality (HR = 0.79; 95% CI, 0.69–0.91), and the association was stronger with any MSA combined with MVPA (hazard ratio [HR] = 0.60; 95% CI, 0.49–0.72). The authors noted that limitations of their review were the heterogenous nature of MSA assessment across the 11 studies and the small number of studies.

Better understanding is needed of the relationship between MSA and risk of death from all causes, cardiovascular disease, or cancer, independent of and in combination with MVPA, to inform public health messaging related to physical activity. Furthermore, because the US population of older adults (≥65) is expected to dramatically increase in the next 10 to 20 years (6), and because most chronic disease occurs in older adults, identifying modifiable factors for disease prevention and longevity will be essential. Although previous studies included a wide age range of adults, some as young as 18, some results suggest that MSA may be more important in older than in younger adults (4). We used data from a large, prospective US-based cohort study (7) of predominantly older adults (≥65) to analyze the association between MSA and mortality, independent of and in combination with MVPA.

## Methods

We examined records on 184,185 men and women who participated in the Cancer Prevention Study-II Nutrition Cohort (CPS-IINC), a prospective study of cancer incidence and mortality established by the American Cancer Society in 1992 (7). Cohort recruitment and characteristics are described elsewhere (7). Briefly, the CPS-IINC is a subgroup of the approximately 1.2 million participants in the CPS-II mortality cohort, which was established in 1982 (8). CPS-IINC participants resided in 21 states with population-based state cancer registries, were aged 50 to 74 in 1992, and completed a mailed questionnaire that assessed demographic, medical, and lifestyle factors, such as physical activity. Beginning in 1997 and every 2 years thereafter, surviving participants were sent a follow-up questionnaire to update lifestyle and medical information. For our analysis, we used the 2001 CPS-IINC survey (N = 143,500 participants), the first year that the survey queried MSA, as the baseline. We excluded people whose questionnaires showed a personal history of cancer, cardiovascular disease, stroke, emphysemsa, or lung disease; were missing body mass index (BMI) (weight in kg/height in m^2^), MVPA, MSA, or smoking status; had a date of death that preceded the date of the survey return; or self-reported poor health (Table 1). After exclusions, our study cohort consisted of 72,462 participants (mean age, 70.2; SD, ≥6.0). The study was approved by Emory University’s institutional review board. 

**Table 1:** Respondents (n = 71,038^a^) Excluded From Analysis, Cancer Prevention Study-II Nutrition Cohort^b^, 2001–2014

Reason for Exclusion	No. of Respondents
**Initial eligible population**	143,500
**Exclusions**	
Personal history of cancer	30,333
Personal history of cardiovascular disease	21,612
Personal history of stroke	4,676
Personal history of emphysema or lung disease	7,473
Missing body mass index	5,121
Missing MVPA or MSA	1,380
Missing smoking status	80
Date of death before date of survey return	3
Self-reported poor health	360
**Final analytic population**	72,462

Abbreviations: MSA, muscle-strengthening activity; MVPA, moderate to vigorous intensity physical activity.

a Excluded from 143,500 participants in the 2001 CPS-IINC survey, the first year that the survey queried MSA, which served as the baseline for our analysis.

b American Cancer Society (7).

CPS-IINC assesses MSA and MVPA with the question, “During the past year, what was the average time per week you spent at the following kinds of activities: walking, jogging/running, lap swimming, tennis or racquetball, bicycling or stationary biking, aerobics/calisthenics, dancing, [or] weight training/resistance exercises?” Responses regarding each activity were none, 1 to 19 minutes, 20 to 59 minutes, 1 hour, 1 to 1.5 hours, 2 to 3 hours, 4 to 6 hours, 7 to 10 hours, or ≥11 hours. MSA was categorized as none, >0 to <1 hours/week, 1 to <2 hours/week, and ≥2 hours/week of weight training or resistance exercise. Summary MVPA metabolic equivalents of task (MET) hours per week were calculated by multiplying the midpoint of each aerobic activity category by the general MET value of each activity (9). To provide conservative summary measures because of the likelihood of overreporting physical activity and the older age of participants, MET values assigned were 3.5 for walking, 7.0 for jogging/running, 7.0 for lap swimming, 6.0 for tennis/racquetball, 4.0 for bicycling/exercise machines, 4.5 for aerobics/calisthenics, and 3.5 for dancing. MVPA was categorized as <7.5 MET hours/week, 7.5 to <15 MET hours/week, and ≥15 MET hours/week. For reference, 7.5 MET hours/week is equal to the minimum recommended MVPA. Although the exact physical activity question in our study was not validated against a criterion measure, it is very similar to that used and validated in the Nurses’ Health Study II, a prospective study with similar participant characteristics, which found a correlation of 0.79 between activity reported on recalls and questionnaire (10). This MVPA measure also demonstrated predicted validity in several other studies in the CPS-IINC cohort (11–15).

Biennial automated linkage of the entire cohort with the National Death Index is used to ascertain deaths in CPS-IINC. For this analysis, deaths were classified by using the Tenth Edition of the International Classification of Disease (ICD-10) (16) for deaths occurring between completion of the 2001 CPS-IINC and December 31, 2014. The primary endpoint in this analysis was all-cause mortality, and secondary endpoints grouped deaths from cardiovascular disease (ICD-10 codes I00–I99 and R96) and cancer (ICD-10 codes C00–C97 and D00–D48).

We used Cox proportional hazards regression to compute HRs and 95% CIs for the association of MSA at baseline and mortality end points in 3 models: model 1) controlled for for sex (male, female) and age (continuous single year), 2) additionally adjusted for other potential confounders including BMI, race, marital status, education, self-reported overall health, aspirin use, smoking status (years since quitting among former smokers, and duration and amount among current smokers), alcohol intake, sitting time while watching television, mobility limitations, and comorbidities (hypertension, type 2 diabetes, and high cholesterol), and 3) all covariates in model 2 with additional adjustment for MVPA. We considered additional potential confounders, such as measures of diet quality. Those that did not result in any change in risk estimates were not included in the final multivariable models. All covariate data were ascertained at baseline at the same time as MSA. Linear and quadratic trends were tested by using the median value of each MSA category, and the quadratic trend *P* value was calculated from the Wald χ^2 ^statistic. All tests for significance were 2-sided, and *P* < .05 was considered significant. We used R version 3.5.2 to conduct all analyses (17).

We conducted several sensitivity analyses. First,we exmained associations restricted to lifelong nonsmokers to rule out the possibility of residual confounding by smoking. Second, we performed analyses restricted to participants who reported no major physical limitations to account for inactive participants who may have been unable to engage in activity and may also have been at higher risk of mortality, because this would lead to bias away from the null. Lastly, although we excluded participants with a history of major chronic conditions, inactive participants possibly had other underlying conditions. Thus, we excluded those who died within the first 2 years of follow-up to minimize possible reverse causation. We examined associations stratified by sex, age, BMI, history of high blood pressure, and MVPA. We used interaction terms between MSA and follow-up time to test the Cox proportional hazards assumption and observed no violations.

## Results

During 13 years of follow-up, 17,750 participants (25% of the cohort) died. At baseline, most participants (79.8% of men and 84.6% of women) reported no MSA, and 5.6% of men and 7.9% of women reported no MVPA. Compared with participants who engaged in MSA, participants who did not engage in any MSA were slightly older, had a higher BMI, were less likely to engage in MVPA, and were more likely to have a higher comorbidity score, some physical limitations, and be nonsmokers and nondrinkers (Table 2).

**Table 2:** Participant Characteristics at Baseline by Hours Of Muscle Strengthening Activity per Week (N = 72,462), Cancer Prevention Study-II Nutrition Cohort^a^, 2001–2014

Characteristic	Muscle-Strenthening Activity, Hours per Week			
	None	>0 to <1	1 to <2	≥2
**Number**	59,952	4,516	4,524	3,470
**Age, y, mean (SD)**	70.49 (6.03)	69.11 (5.90)	69.03 (5.68)	68.81 (5.60)
**Body mass index (height in kg/weight in m2), mean (SD)**	26.46 (4.48)	25.38 (4.10)	25.25 (3.85)	25.51 (3.85)
**Race, n (%)**				
White/white Hispanic	58,434 (97.5)	4,397 (97.4)	4,415 (97.6)	3,380 (97.4)
Black/black Hispanic/black other	1,518 (2.5)	119 (2.6)	109 (2.4)	90 (2.6)
**Marital status, n (%)**				
Married	43,100 (71.9)	3,468 (76.8)	3,519 (77.8)	2,732 (78.7)
Other	16,852 (28.1)	1,048 (23.2)	1,005 (22.2)	738 (21.3)
**Education, n (%)**				
High school diploma or less	20,407 (34.0)	740 (16.4)	713 (15.8)	550 (15.9)
Some college	17,121 (28.6)	1,213 (26.9)	1,136 (25.1)	875 (25.2)
College graduate or higher	22,424 (37.4)	2,563 (56.8)	2,675 (59.1)	2,045 (58.9)
**Self-reported overall health, n (%)**				
Excellent	8,321 (13.9)	955 (21.1)	1,174 (26.0)	1,029 (29.7)
Very good	25,411 (42.4)	2,076 (46.0)	2,130 (47.1)	1,615 (46.5)
Good	21,024 (35.1)	1,226 (27.1)	1,021 (22.6)	670 (19.3)
Fair	3,671 (6.1)	155 (3.4)	90 (2.0)	72 (2.1)
**Aspirin use, n (%)**				
None	26,114 (48.1)	1,913 (42.4)	1,906 (42.1)	1,507 (43.4)
<15 days/month	7,418 (13.7)	646 (14.3)	597 (13.2)	411 (11.8)
**≥**15 days/month	20,764 (38.2)	1,644 (36.4)	1,731 (38.3)	1,310 (37.8)
**Smoker, n (%)**				
Never	30,689 (51.2)	2,167 (48.0)	2,052 (45.4)	1,534 (44.2)
Former, <20 y since quit	7,390 (12.3)	517 (11.4)	566 (12.5)	468 (13.5)
Former, 20 to <30 y since quit	5,489 (9.2)	484 (10.7)	516 (11.4)	412 (11.9)
Former, ≥30 y since quit	13,178 (22.0)	1,215 (26.9)	1,247 (27.6)	964 (27.8)
Current, smoked <15 cigarettes/d for <45 y	470 (0.8)	27 (0.6)	24 (0.5)	23 (0.7)
Current, smoked, ≥15 cigarettes/d for <45 y	333 (0.6)	8 (0.2)	10 (0.2)	4 (0.1)
Current, smoked <15 cigarettes/d for ≥45 y	600 (1.0)	23 (0.5)	33 (0.7)	16 (0.5)
Current, smoked, ≥15 cigarettes/d for ≥45 y	700 (1.2)	16 (0.4)	18 (0.4)	18 (0.5)
**Alcohol use, n (%)**				
Not current user	22,652 (37.8)	1,260 (27.9)	1,161 (25.7)	927 (26.7)
<1 drink/wk	3,818 (6.4)	313 (6.9)	302 (6.7)	192 (5.5)
1–6 drinks/wk	13,700 (22.9)	1,304 (28.9)	1,371 (30.3)	1,018 (29.3)
1 drink/d	5,842 (9.7)	644 (14.3)	678 (15.0)	534 (15.4)
**≥**2 drinks/d	4,626 (7.7)	396 (8.8)	459 (10.1)	367 (10.6)
**Moderate-to-vigorous physical activity or metabolic equivalent, h/wk, n (%)**				
<7.5	27,868 (46.5)	1,246 (27.6)	739 (16.3)	457 (13.2)
7.5 to <15	13,144 (21.9)	1,091 (24.2)	1,099 (24.3)	586 (16.9)
**≥**15	18,940 (31.6)	2,179 (48.3)	2,686 (59.4)	2,427 (69.9)
**Sitting time while watching television, h/wk, n (%)**				
<7	22,272 (37.1)	1,877 (41.6)	1,774 (39.2)	1,290 (37.2)
7–30	25,993 (43.4)	1,916 (42.4)	2,133 (47.1)	1,668 (48.1)
>30	2,970 (5.0)	173 (3.8)	139 (3.1)	134 (3.9)
**Mobility limitations, n (%)**				
None	28,544 (47.6)	2,608 (57.8)	2,889 (63.9)	2,334 (67.3)
Some	19,658 (32.8)	1,205 (26.7)	1,033 (22.8)	666 (19.2)
Major	4,326 (7.2)	217 (4.8)	185 (4.1)	143 (4.1)
**Comorbidity score,^b^ n (%)**				
0	13,742 (22.9)	1,210 (26.8)	1,250 (27.6)	943 (27.2)
1	23,141 (38.6)	1,849 (40.9)	1,792 (39.6)	1,442 (41.6)
2	19,203 (32.0)	1,256 (27.8)	1,278 (28.2)	939 (27.1)
3	3,866 (6.4)	201 (4.5)	204 (4.5)	146 (4.2)

a American Cancer Society (7). Some percentages may not add to 100% because of missing information.

b Reported personal history of high blood pressure, type 2 diabetes, or high cholesterol = 1 point each (range 0–3).

Associations between MSA and mortality appeared to be nonlinear, and additionally adjusting for MVPA in multivariable models did not appreciably change any risk estimates (Table 3). After adjusting for MVPA and other factors, engaging in >0 to <2 hours/week of MSA compared with no MSA was associated with a lower risk of all-cause mortality (>0 to <1 hr/wk: HR = 0.88; 95% CI, 0.82–0.94); 1 to <2 hr/wk, HR = 0.90; 95% CI, 0.84–0.97), but engaging in ≥2 hr/wk was not (quadratic trend *P* value, <.001). Associations were similar, albeit not significant, for cancer mortality. Engaging in >0 to <1 hr/wk of MSA was associated with 19% lower risk (95% CI, 0.71–0.92) of cardiovascular disease mortality, but greater time spent in MSA was not (quadratic trend *P* value, .005).

**Table 3:** Cox Proportional Hazard Ratios of All-Cause, Cancer, and Cardiovascular Disease Mortality, by Hours Per Week Of Muscle-Strengthening Physical Activity, Participants (N = 72,462), Cancer Prevention Study-II Nutrition Cohort,^a^ 2001–2014

Mortality^b^	MSA, Hours per Week	No. Events	Person Years	Model 1^c^	Model 2^d^	Model 3^e^
**All causes**	—	15,671	706,525	1 [Reference]		
	>0 to <1 h	852	55,001	0.78 (0.73–0.83)	0.86 (0.80–0.92)	0.88 (0.82–0.94)
	1 to <2 h	829	55,322	0.77 (0.71–0.82)	0.86 (0.81–0.93)	0.90 (0.84–0.97)
	≥2 h	682	42,355	0.82 (0.76–0.89)	0.96 (0.89–1.04)	1.01 (0.93–1.09)
	Linear trend* P* value	—	—	<.001	.002	.048
	Quadratic trend *P* value	—	—	<.001	<.001	<.001
**Cancer**	None	4,281	706,525	1 [Reference]		
	>0 to <1 h	262	55,001	0.83 (0.73–0.94)	0.91 (0.80–1.03)	0.92 (0.81–1.04)
	1 to <2 h	270	55,322	0.84 (0.75–0.96)	0.92 (0.81–1.04)	0.94 (0.83–1.06)
	≥2 h	225	42,355	0.90 (0.79–1.03)	1.00 (0.87–1.14)	1.02 (0.89–1.17)
	Linear trend *P* value	—	—	.002	.34	.60
	Quadratic trend *P* value	—	—	.01	.10	.11
**Cardiovascular disease**	None	5,053	706,525	1 [Reference]		
	>0 to <1 h	241	55,001	0.70 (0.61–0.79)	0.79 (0.69–0.90)	0.81 (0.71–0.92)
	1 to <2 h	270	55,322	0.81 (0.72–0.92)	0.93 (0.82–1.05)	0.98 (0.86–1.10)
	≥2 h	206	42,355	0.81 (0.71–0.94)	0.98 (0.85–1.13)	1.03 (0.90–1.19)
	Linear trend *P* value	—	—	<.001	.16	.71
	Quadratic trend *P* value	—	—	<.001	.003	.005

Abbreviations: —, not applicable; HR, hazard ratio; MSA, muscle strengthening activity.

a American Cancer Society (7).

b Values are hazard ratio (95% confidence interval).

c Model 1 is adjusted for sex and age (single year).

d Model 2 is adjusted for sex and age, and additionally adjusted for BMI, survey type (long/short), education, self-reported overall health, smoking duration and intensity, alcohol use, marital status, work status, TV sitting time, aspirin use, and comorbidity score (reported personal history of high blood pressure, type 2 diabetes, and high cholesterol).

e Model 3 includes all covariates from Model 2 and additionally adjusts for aerobic moderate-to-vigorous physical activity (<7.5, 7.5 - <15, ≥15 h/wk).

We observed a mortality benefit with moderate amounts of MSA (>0 to <2 hr/wk) across all levels of MVPA (interaction *P* =.79). We found no evidence of effect modification by sex, age, BMI, or history of hypertension. Sensitivity analyses restricted to lifelong nonsmokers or to participants with no physical limitations or after excluding the first 2 years of follow-up were also virtually unchanged from those in the main analyses.

## Discussion

In our analysis of data from our large prospective study, engaging in less than 2 hours per week of MSA was associated with lower risk of mortality from all-causes, cardiovascular disease, and cancer compared with engaging in no MSA, independent of MVPA. We found no mortality benefit with higher amounts of MSA. Similarly, most prior studies found that engaging in any MSA was associated with lower mortality risk compared with engaging in none (4,5), independent of MVPA. Our results are also consistent with the one meta-analysis of MSA and mortality that reported a J-shaped association (5). That study found that engaging in up to 2 sessions of MSA per week compared with none was associated with lower mortality risk (HR = 0.79; 95% CI, 0.66–0.95), but engaging in more than 2 sessions was no longer associated (≥5 sessions vs none, HR = 1.07; 95% CI, 0.90–1.26). One study proposed that the lack of a linear dose–response relationship, especially for cardiovascular disease mortality, may be due to increased arterial stiffness with greater amounts of high intensity strength training (18). However, few studies have examined the association between cardiovascular disease mortality and MSA to date (5). Additional studies are needed.

The association between MSA and cancer mortality also requires confirmation, because fewer than 5 studies have examined this endpoint (4,5) and with somewhat mixed results. Our results and those from the meta-analysis (5) found inverse associations, albeit nonsignificant, whereas the pooled analysis (4) found a stronger association (HR = 0.66; 95% CI, 0.48–0.92 with adherence to the MSA guideline [MSA twice a week] vs no MSA).

Our study found no evidence of effect modification by MVPA, and MSA appears to be beneficial regardless of engaging in MVPA. A pooled analysis of 11 studies similarly found only a modest difference when considering both MSA and MVPA (HR = 0.79; 95% CI, 0.66–0.94 when adhering to only the MSA guideline; HR = 0.70; 95% CI, 0.57–0.87 when adhering to both MSA and MVPA guidelines). In contrast, the only meta-analysis to date (5) reported that engaging in any MSA was associated with a 21% lower risk of mortality (HR = 0.79; 95% CI, 0.69–0.91), but the association was stronger when engaging in any MSA combined with MVPA (HR = 0.60; 95% CI, 0.49–0.72).

Several possible mechanisms may explain an inverse association between MSA and mortality risk. MSA improves glucose homeostasis, and is associated with lower risk of type 2 diabetes and cardiometabolic risk factors such as hypertension (2,3). Additionally, MSA has been associated with lower risk of having multiple disorders (19), which would be associated with risk of premature death from all causes. Various musculoskeletal and physical functions benefit from MSA, which might contribute to overall improved longevity, such as increases in muscle mass and muscle strength, both of which have been shown to be associated with lower mortality risk (20,21).

Strengths of our study were its large sample size and the prospective design of CPS-IINC and its ability to control for many potential confounders. Limitations were the lack of racial/ethnic diversity in the study population (approximately 98% of the study population was non-Hispanic white). Other limitations were the lack of information on types and intensities of MSA, lack of information on past or long-term engagement in MSA, the use of self-reported physical activity information, and the relatively low percentage of participants engaging in any MSA. Lastly, an additional limitation of our study was the lack of information on occupational physical activity. However, given the older age of the population, most participants were retired, and past employment was largely sedentary occupations or homemaking. Previous studies lacked broad consistency in how MSA was queried, which limits the ability to interpret results across studies. For example, previous studies varied in timeframe of exposure assessment (ranging from the previous 4 weeks to the past year), in the level of detail on intensity, and in categories of duration of exposure (4,5).

Engaging in moderate amounts of MSA is associated with lower all-cause mortality, independent of MVPA. Overall, 23.2% of US adults report engaging in MSA at least twice a week, and this percentage drops to 16.4% for adults aged 65 to 74 and 10.2% for older adults (22); thus, it is important to reinforce recommendations to engage in MSA for overall health, especially among older adults. Additional research is needed to better understand the types, amount, and intensity of strength-training activities that would confer the greatest health benefits.
